# Innate Immune Cells in Pressure Overload-Induced Cardiac Hypertrophy and Remodeling

**DOI:** 10.3389/fcell.2021.659666

**Published:** 2021-07-23

**Authors:** Xin Liu, Guo-Ping Shi, Junli Guo

**Affiliations:** ^1^Department of Medicine, Brigham and Women’s Hospital and Harvard Medical School, Boston, MA, United States; ^2^Department of Cardiology, Renmin Hospital of Wuhan University, Cardiovascular Research Institute, Wuhan University, Hubei Key Laboratory of Cardiology, Wuhan, China; ^3^Hainan Provincial Key Laboratory for Tropical Cardiovascular Diseases Research & Key Laboratory of Emergency and Trauma of Ministry of Education, Institute of Cardiovascular Research of the First Affiliated Hospital, Hainan Medical University, Haikou, China

**Keywords:** pressure overload, hypertrophy, fibrosis, innate immune cell, cardiomyocyte, cardiac fibroblast

## Abstract

Pressure overload and heart failure are among the leading causes of cardiovascular morbidity and mortality. Accumulating evidence suggests that inflammatory cell activation and release of inflammatory mediators are of vital importance during the pathogenesis of these cardiac diseases. Yet, the roles of innate immune cells and subsequent inflammatory events in these processes remain poorly understood. Here, we outline the possible underlying mechanisms of innate immune cell participation, including mast cells, macrophages, monocytes, neutrophils, dendritic cells, eosinophils, and natural killer T cells in these pathological processes. Although these cells accumulate in the atrium or ventricles at different time points after pressure overload, their cardioprotective or cardiodestructive activities differ from each other. Among them, mast cells, neutrophils, and dendritic cells exert detrimental function in experimental models, whereas eosinophils and natural killer T cells display cardioprotective activities. Depending on their subsets, macrophages and monocytes may exacerbate cardiodysfunction or negatively regulate cardiac hypertrophy and remodeling. Pressure overload stimulates the secretion of cytokines, chemokines, and growth factors from innate immune cells and even resident cardiomyocytes that together assist innate immune cell infiltration into injured heart. These infiltrates are involved in pro-hypertrophic events and cardiac fibroblast activation. Immune regulation of cardiac innate immune cells becomes a promising therapeutic approach in experimental cardiac disease treatment, highlighting the significance of their clinical evaluation in humans.

## Introduction

Pressure overload refers to the left ventricular (LV) pressure overload caused by aortic stenosis, hypertension, and coarctation of the aorta, and right ventricular (RV) pressure overload triggered by pulmonary stenosis and pulmonary hypertension, leading to cardiac hypertrophy and fibrosis (Berk et al., 2007). Such remodeling exhibits extensive morphological changes, including cardiomyocyte mass increase, sarcomere rearrangement, and extracellular matrix (ECM) deposition in cardiac interstitial or perivascular regions (Schiattarella and Hill, 2015). At the early stage, cardiac hypertrophy and ventricular dilatation are compensatory responses to pressure overload stimuli (Schiattarella and Hill, 2015). Yet, chronic pressure overload stimulation increases heart wall thickness, fibrotic protein deposition, and inflammatory cell infiltration, which give rise to myocardial compliance reduction and hemodynamic dysfunction. At the late stage, sustained pressure overload results in life-threatening heart failure (HF) accompanied by serious adverse events including respiratory failure and cardiac arrest (Shimizu and Minamino, 2016). Therefore, pressure overload-induced cardiac hypertrophy has been used as an experimental model to study human HF with preserved ejection fraction HFpEF (Mishra and Kass, 2021). One popular theory in HFpEF patients is the established pro-inflammatory state with elevated levels of CD3^+^ T cells, CD68^+^ macrophages and monocytes, or total CD45^+^ leukocytes in myocardial biopsy specimens from these patients (Westermann et al., 2011; Hahn et al., 2020). Further evidence of inflammatory conditions in HFpEF patients came from elevated blood inflammatory biomarkers, including interleukin-1β (IL1β), IL6, IL10, immunoglobulin-like transcript 6, tumor necrosis factor-α (TNF-α), TNF-receptor, matrix metalloproteinases (MMP-7), MMP-9, and myeloperoxidase (Chirinos et al., 2020). Similarly, the activation of immune cells, especially innate immune cells, orchestrates pressure overload-induced cardiac hypertrophy and fibrosis. Activated immune cells produce high levels of cytokines that induce cardiomyocyte hypertrophy, such as TNF-α, IL1β, and IL6. Increased TNF-α expression was associated with cardiac hypertrophy (You et al., 2018). TNF-α deficiency blunted pressure overload-induced cardiac hypertrophy (Sun et al., 2007). Similarly, IL1β deficiency also protected mice from pressure-mediated hypertrophy (Honsho et al., 2009) and deficiency of IL6 suppressed angiotensin-II (Ang-II)-induced cardiomyocyte hypertrophy (Chen et al., 2017). In addition, inflammation is also critical in the initiation, propagation, and development of cardiac fibrosis. Immune signaling triggers the accumulation, proliferation, and activation of fibroblasts by producing proteases that participate in matrix metabolism, fibrogenic mediator, and ECM protein secretion that exert contact-dependent actions on fibroblast phenotype (Dostal et al., 2015). Herein, we summarize the current understanding of innate immune cells with a focus on pressure overload-induced cardiac hypertrophy. We highlight the cross talk between innate immune cells and cardiac remodeling to propose a therapeutic potential to target these cells in humans.

### Pressure Overload-Induced Cardiac Hypertrophy and Fibrosis

Use of experimental models makes it possible to study the molecular and cellular mechanisms by which pressure overload induces heart hypertrophy. The murine transverse aortic constriction (TAC) model was first validated by Rockman et al. (1991), and has since been commonly used to mimic human clinical aortic stenosis with high LV afterload. Chronic subjection to Ang-II in mice imitates chronic systemic hypertension due to neurohumoral activation of the renin–angiotensin–aldosterone system (Paul et al., 2006). The murine model of pulmonary artery constriction (PAC) generates RV hypertrophy and fibrosis following pulmonary artery hypertension (Braun et al., 2003). Another model that mimics pressure overload is aldosterone analog deoxycorticosterone acetate (DOCA) accompanied by unilateral nephrectomy and high-salt diet. Chronic subjection of DOCA promotes hypertension and subsequent development of HF in mice (Silberman et al., 2010; Lovelock et al., 2012). Hypertension from the DOCA model could be divided into two phases: an initial peak in blood pressure at an early phase and sustained hypertension and cardiac remodeling at the late phase (Alex and Frangogiannis, 2018). Together, pressure overload-induced hypertrophy in experimental animals displayed enhanced systolic and diastolic blood pressures, increased cardiac mass, and eventually elevated cardiac fibrosis.

### Innate Immune Cells

**FIGURE 1:**
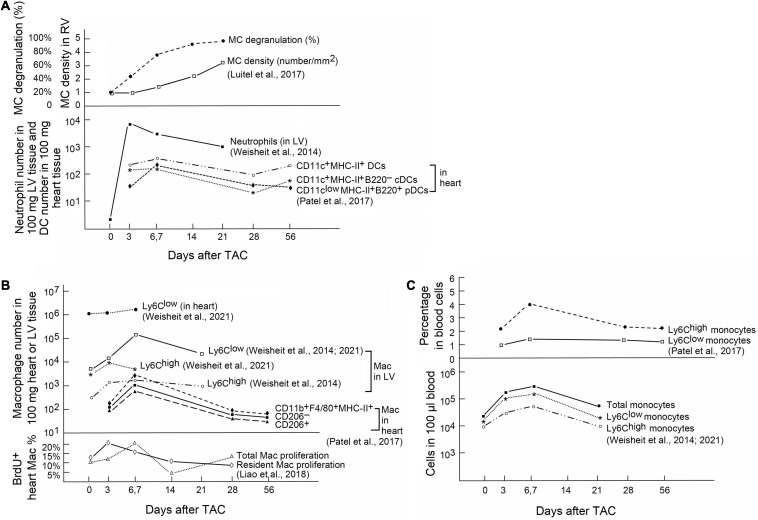
Innate immune cells in heart and blood from mice after pressure overload-induced cardiac hypertrophy. **(A)** Upper: Toluidine blue staining detected MC density and percentage of degranulated MCs in RV from mice with PAC-induced pressure overload. Bottom: FACS determined the absolute number of neutrophils and different DCs in 100 mg heart or LV tissues from mice after TAC-induced pressure overload. **(B)** Upper: FACS analysis of different macrophage subsets in 100 mg heart or LV tissues from mice with TAC-induced pressure overload. Bottom: BrdU staining followed by FACS analysis to detect proliferation of heart-resident and total macrophages in TAC-treated mice. **(C)** Upper: Percentage of different monocyte subsets in total blood cells from TAC-treated mice. Bottom: Absolute numbers of different monocyte subsets in 100 μl of blood from TAC-treated mice. Graphs were generated by grouping the results from different studies. References are indicated.

Innate immune cells are a group of cells that sense signals from pathogens and endogenous sources and can be triggered to give an immediate and non-specific response. Pathogenic bacteria, viruses, fungi, and parasites share small molecular motifs, known as pathogen-associated molecular patterns (PAMPs). These patterns could be identified by pattern recognition receptors (PRR) and toll-like receptors (TLRs) in resident immune cells or myocardial cells (Takeuchi and Akira, 2010). After cardiac injury, autoimmunity leads to cardiac cell apoptosis or necrotic cell death followed by productions of damage-associated molecular patterns (DAMPs) (Frangogiannis, 2014; Shinde and Frangogiannis, 2014). The main DAMPs include IL1α, galectin-3, high mobility box group1 protein (HMGB1), S100 protein, and heat shock protein (HSP) (Seong and Matzinger, 2004) that bind to IL1 receptor-1 (Di Paolo and Shayakhmetov, 2016), CD45 and CD71 (Stillman et al., 2006), TLR4 and receptor for advanced glycation end (RAGE) (Yang et al., 2020), RAGE (Leclerc et al., 2009), and G-protein cell receptors (Streicher, 2019), respectively, to exert their pathophysiological activities. A large body of evidence shows that innate immune cells play a considerable role in the development of cardiac hypertrophy and fibrosis (Frieler and Mortensen, 2015). Single-cell RNA sequencing from recent studies demonstrated that the majority innate immune cell subpopulations, including mast cells (MCs), monocytes and macrophages, neutrophils, dendritic cells (DCs), eosinophils (EOS), and invariant natural killer T (iNKT) cells underwent extensive activation in pressure overload-induced HF in mice (Martini et al., 2019). Different innate immune cells accumulate in hearts at different time points after pressure overload injury. On heart sections from mice with PAC-induced pressure overload, toluidine blue staining revealed increase of MCs in mouse RV myocardium. Cardiac MCs peaked at 21 days after the injury. MC degranulation was also increased rapidly and reached to about 80% within a week after cardiac injury (Luitel et al., 2017; Figure 1A, upper panel). In contrast, neutrophils are probably the first immune cells that come to the hearts in response to pressure overload injury. In TAC-treated mice, LV neutrophils peaked in 3 days after injury and remained high in 3 weeks or longer (Weisheit et al., 2014; Figure 1A, bottom panel). DCs acted differently. In TAC-induced hypertrophy in mice, FACS analysis illustrated that CD11c^*low*^MHC-II^+^B220^+^ plasmacytoid DCs (pDCs) peaked in 1 week after the injury. In contrast, CD11c^+^MHC-II^+^B220^–^ conventional DCs (cDCs) accumulated in the heart tissue in a biphasic manner, with peaks at both early (1 week) and late (8 weeks) phases after TAC injury (Patel et al., 2017; Figure 1A, bottom panel). Macrophages and monocytes are probably the most studied cell types in hypertrophic hearts. In TAC-induced mouse hypertrophic hearts, FACS analysis showed that the CD206^+^ or CD206^–^ macrophages or CD11b^+^F4/80^+^MHC-II^+^ macrophages peaked at 6–7 days after TAC injury in mice (Patel et al., 2017; Figure 1B, upper panel). A separate study showed slightly different results. Ly6C^*low*^ macrophages in heart or LV tissues peaked at 7 days after TAC injury, but Ly6C^*high*^ macrophages in LV tissues peaked at 3 days after TAC injury (Weisheit et al., 2014, 2021; Figure 1B, upper panel). Bromodeoxyuridine (BrdU) FACS analysis showed that the proliferation of cardiac resident macrophages peaked at 3 days after TAC injury, whereas the proliferation of total macrophages peaked at 7 days after TAC surgery (Figure 1B, bottom panel) (Liao et al., 2018). In blood, both Ly6C^*high*^ and Ly6C^*low*^ monocytes peaked at 7 days after TAC surgery and then went back to the baseline in 3 to 4 weeks (Figure 1C, upper and bottom panels) (Weisheit et al., 2014, 2021; Patel et al., 2017). Yet, limited information is available from many other innate immune cells regarding their cardiac infiltration. Time cause differences in cardiac infiltration of these innate immune cells suggest their differences in cardioprotective or cardiodestructive functions in pressure overload-induced hypertrophic heart.

## Mast Cells

**FIGURE 2:**
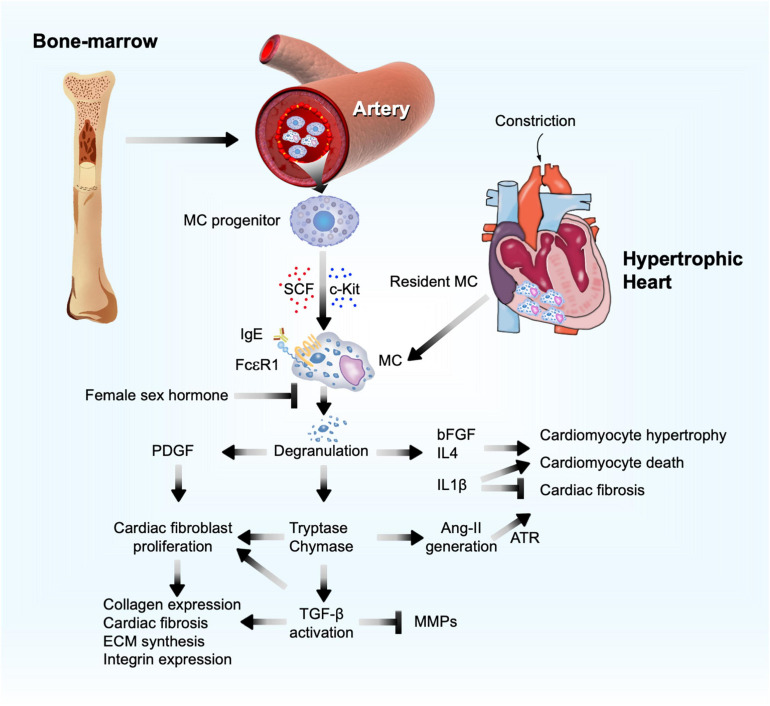
Mast cell function in pressure overload-induced cardiac hypertrophy and fibrosis. Bone marrow MC progenitor-derived and heart-resident MCs get activated and release PDGF, proteases (chymase and tryptase), and cytokines to affect cardiomyocyte hypertrophy, cardiac cell death, and cardiac fibroblast ECM and fibrotic protein syntheses, as a mechanism to promote cardiac hypertrophy and fibrosis.

Mast cells were first linked to cardiac fibrosis more than 50 years ago following the observation that these cells accumulated in the endocardial fibrotic region from an autopsy series of 672 cases (Fernex and Sternby, 1964). Since then, the bulk of evidence has shown that myocardial MCs increased after cardiac injury from multiple etiologies. MCs are considered non-circulating cells and developed only from bone-marrow-derived precursors. Driven by stem cell factor (SCF) and its receptor c-kit, MC progenitors are recruited through the blood stream and target the terminal tissues where MCs differentiate and mature. Cardiac MCs possess IgE receptor FcεRI, TNF-α receptor I, and C5a complement receptor (Ito et al., 1993; Fureder et al., 1995; Patella et al., 1995). After activation of these receptors, cardiac MCs are capable of degranulating and releasing preformed mediators from their granules, although MCs can also *de novo* synthesize and secrete their intracellular mediators without granule involvement (Kandere-Grzybowska et al., 2003; Zhang et al., 2012). MC granules contain excessive specific substances (e.g., histamine), proteases (e.g., tryptase and chymase), non-MC-specific proteases (cathepsin G), amines (serotonin and dopamine), cytokines (TNF-α, IL4, and IL5), and growth factors (SCF and fibroblast growth factor [bFGF]) (Wernersson and Pejler, 2014; Figure 2).

### MC Function in Pressure Overload-Induced Cardiac Hypertrophy and Fibrosis

Several studies suggested MC involvement in pressure overload-induced cardiac hypertrophy and HF. In a study of 86 aortic valve stenosis patients who underwent valve replacement and 17 control subjects, cardiac expression of cathepsin G, a common MC protease, was associated with areas rich in MCs. Cathepsin G expression correlated with heart collagen I and III contents (Helske et al., 2006). In hypertrophic and failing human hearts, both MC contents and MC chymase expression were increased (Batlle et al., 2006). Animal studies also reported high MC density in the myocardium from different animal models of pressure overload-induced cardiac hypertrophy. MCs were found increased by more than threefold in rat RV myocardium following PAC injury, resulting in hemodynamic dysfunction, RV enlargement, and fibrosis (Olivetti et al., 1989). In a mouse model of PAC-induced pressure overload and RV hypertrophy, the number of MCs and their degranulation increased by two- to threefold in RV after PAC (Luitel et al., 2017; Figure 1A, upper panel). In TAC-induced hypertrophic mice, MCs were also increased by over twofold in the atrium (Liao et al., 2010). In 36-week-old spontaneously hypertensive rats with established LV hypertrophy, increase of LV MC number occurred together with increased interstitial and perivascular fibrosis and hydroxyproline concentration, an indication of increased collagen contents. Univariate regression test showed that MC number in LV correlated significantly with collagen volume fraction (*r* = 0.87, *p* < 0.001; Panizo et al., 1995).

Mast cell activation and mediator release have also been proven essential in experimental cardiac hypertrophy. After TAC treatment, all rats with or without ovariectomization developed cardiac hypertrophy and myocardium MC accumulation. Yet, ovariectomized rats showed increased myocardium collagen fraction, with much greater release of myocardial chymase and active transforming growth factor-β1 (TGF-β1), and even increased plasma chymase levels. Estrogen (17β-estradiol) replacement, chymase inhibition with chymostatin, or MC stabilization with nedocromil reduced pressure overload-induced ventricular hypertrophy in ovariectomized rats, along with reduced myocardial chymase, TGF-β1, and MC contents, and plasma chymase levels (Li et al., 2015). It seems that estrogen blocked MC function in this study (Figure 2). Yet, prior studies showed that female sex hormones, estradiol and progesterone, activate MCs (Zierau et al., 2012). Women at reproductive age or receiving sex hormone therapy showed increased risk of asthma (Zierau et al., 2012). Therefore, estrogen (17β-estradiol) replacement-mediated reduction of pressure overload-induced hypertrophy in ovariectomized rats may be independent of MC function. For example, estrogen induces β-adrenergic receptor expression (Wagner et al., 1979) and directly targets cardiomyocytes and cardiac fibroblasts that also express estrogen receptor (Grohe et al., 1997).

Mast cell-deficient W/Wv mice have been used widely to examine their roles in different hypertrophic models. From suprarenal abdominal aortic constriction (SAC)-induced pressure overload, wild-type (WT) mice showed worse LV performance with decompensated hypertrophy and pulmonary congestion at 4 weeks after constriction than W/Wv mice. In contrast, throughout 15 weeks after constriction, W/Wv mice still showed preserved LV performance, and neither their decompensation of cardiac function or pulmonary congestion was observed. In addition, perivascular fibrosis and MC chymase upregulation were less apparent in W/Wv mice (Li et al., 2008). In TAC-treated hypertrophic mice, programmed electrical stimulation to the right atrium under Langendorff perfusion induced atrial fibrillation (AF). In these mice, TAC induced MC infiltration and fibrosis in the atrium and enhanced AF susceptibility. MC stabilization with cromolyn or reconstitution of bone marrow from W/Wv mice did not change echocardiographic parameters of LV hypertrophy and systolic functions, but markedly reduced AF episode incidence and duration under Langendorff perfusion and fibrotic changes in the atrium. In SAC-induced pressure overload in mice, MC deficiency in W/Wv mice reduced myocardium MC chymase upregulation and perivascular fibrosis and prevented cardiac function decompensation at 15 weeks after pressure overload (Hara et al., 2002).

### MC Molecular and Cellular Mechanisms

Mast cells modulate cardiomyocyte hypertrophy and cardiac fibrosis by secreting inflammatory mediators. MC-derived bFGF and TGF-β1 regulate cardiomyocyte growth and death. MC mediators also regulate cardiac fibrosis progression or regression. For example, MCs release pro-inflammatory IL1, IL6, and interferon (IFN)-γ; anti-inflammatory IL10 and IL13; and pro-fibrotic TGF-β, vascular endothelial growth factor (VEGF), platelet-derived growth factor (PDGF), anti-fibrotic IL33, and prostaglandin D2 (PGD2) (Lefrançais et al., 2014; Overed-Sayer et al., 2014a,b; Janicki et al., 2015; Mukai et al., 2018). MC-derived tryptase and chymase are proteases that convert TGF-β from a latent form to an active form, an essential fibrotic growth factor during the development of cardiac fibrosis by stimulating myofibroblast trans-differentiation (Desmoulière et al., 1993), promoting ECM protein synthesis, increasing integrin expression (Kong et al., 2018), and acting as an inhibitor of ECM degradation by matrix metalloproteinases (MMP) (Baricos et al., 1999; Figure 2). MC tryptase can directly induce fibroblast differentiation independent of TGF-β (Abe et al., 1998; Akers et al., 2000; Figure 2). In TAC-induced cardiac hypertrophy in 5-week old Sprague Dawley rats, tryptase inhibition with nafamostat mesilate (5 mg/kg/day) reduced the ratio of LV weight to tibia length (TL) and plasma chymase levels. Yet, there was no significant difference in collagen volume fraction and hemodynamic indexes between TAC-treated rats and those that also received nafamostat mesilate (Li et al., 2016). Chymase is an angiotensin-converting enzyme (ACE) that generates Ang-II (Caughey et al., 2000; Li et al., 2004; Takai et al., 2004; Miyazaki et al., 2006; Bradding and Pejler, 2018). Ang-II, a well-recognized clinical determinant in reversing maladaptive cardiac hypertrophy, induces cardiomyocyte enlargement and accelerates fibroblast differentiation through its surface angiotensin receptor (ATR) (Lijnen et al., 2001; Ainscough et al., 2009) (Figure 2). In dogs with tachycardia-induced HF, chymase inhibitor SUNC8257 (10 mg/kg, orally twice a day) significantly decreased MC density, cardiac Ang-II expression, and collagen-type I, III, and TGF-β mRNA levels (Matsumoto et al., 2003). Several studies showed that Ang-II production from MC chymase played a negligible role in blood pressure. In spontaneous hypertensive rats, only ACE inhibitor and ATR blocker, but not chymase inhibitor, displayed anti-hypotensive effect (Kirimura et al., 2005). Both systolic and diastolic blood pressures did not differ between WT and W/Wv mice (Li et al., 2004).

Platelet-derived growth factor-α receptor signaling is required for cardiac fibroblast maintenance and activation. Deficiency of PDGF-α receptor expression or inhibition of PDGF-α receptor signaling led to cardiac fibroblast loss in mouse heart and cardiac fibroblast apoptosis in culture (Ivey et al., 2019). In co-cultures of neonatal rat cardiomyocytes or fibroblasts with mouse MCs, cardiomyocytes and cardiac fibroblasts induced MC expression of PDGF-A. In turn, MC-derived PDGF-A promoted cardiac fibroblast activation and collagen expression (Liao et al., 2010). Antibody-mediated neutralization of PDGF-α receptor alleviated the AF inducibility and fibrosis in TAC-induced hypertrophic mice (Liao et al., 2010). MC granules contain bFGF and IL1β that may also promote cardiomyocyte enlargement and remodeling after MC activation by IgE *in vivo* or *in vitro* (Figure 2). MCs are the major source of cardiac bFGF (Shiota et al., 2003). While the 18-kDa bFGF acts in adaptive trophic response, the 34-kDa high-molecular-weight bFGF exacerbates hypertrophy and contributes to cardiac cell death, thereby driving the myocardium towards a maladaptive phenotype (Kardami et al., 2004). In contrast, IL1β can be pleiotropic in hypertrophic heart. IL1β induced cardiomyocyte growth and hypertrophy, but reduced the growth of cultured cardiac fibroblasts (Palmer et al., 1995). Therefore, in pressure overload-induced hypertrophic mice, IL1β deficiency reduced heart weight (HW), cardiomyocyte size, and LV ejection fraction (EF), but greatly increased interstitial fibrosis. Mechanistic studies showed that pressure overload or mechanical stretch induced cardiac fibroblast release of IL1β to induce insulin-like growth factor-1 (IGF-1) production *via* the JAK/STAT signaling pathway. IL1β deficiency enhanced cardiomyocyte apoptosis with concurrent increase of c-Jun N-terminal kinase (JNK) activation and caspase-3 activities. IGF-1 replacement or JNK inhibitor blocked these adverse activities of IL1β deficiency (Honsho et al., 2009). Patients with longstanding pulmonary arterial hypertension (PAH) experienced pressure overload in the RV. Therefore, serum IL1β levels were elevated in these patients (Soon et al., 2010). PAH patients receiving 100 mg subcutaneous anakinra, a recombinant IL1 receptor antagonist, showed reduced plasma C-reactive protein and IL6 and significant improvement of HF symptoms (Trankle et al., 2019). In obese patients, both short-term (2 days) and long-term (4 weeks) treatment of anakinra reduced systolic blood pressure by 4–5 mmHg with decreased stroke systemic vascular resistance index and peripheral vascular resistance (Urwyler et al., 2020).

### Therapeutic Potential of Targeting MCs

Tryptase and chymase inhibitors as well as MC stabilizers may become important regimens in alleviating pressure overload-induced cardiac hypertrophy and fibrosis. MC membrane stabilizers prevent MC degranulation and intracellular granule release, which decreases their interactions with cardiomyocytes, fibroblasts, or other cardiac inflammatory infiltrates following cardiac injury. From 8-week-old rats with spontaneous hypertension, treatment of MC stabilizer nedocromil (30 mg/kg/day) for 12 weeks reduced macrophage infiltration, normalized myocardium MC tryptase level, and prevented LV fibrosis, independent of hypertrophy and blood pressure, although myocardium MC content remained high. Spontaneous hypertension also increased myocardial TNF-α, IFN-γ, and pro-fibrotic IL4, but decreased myocardial IL6 and IL10. Treatment with nedocromil significantly reversed these cytokine profiles. In cultured cardiac fibroblasts from hypertensive rats, tryptase induced fibroblast proliferation and collagen synthesis (Levick et al., 2009). In pressure overload-induced cardiac fibrosis mice, MCs are an essential source of IL4 (Figure 2). MC stabilization with cromolyn reduced MC degranulation, IL4 expression, cardiac fibrosis, and infiltration of interstitial fibroblasts and macrophages in the fibrosis regions (Kanellakis et al., 2012). Similarly, administration of a MC stabilizer tranilast prevented the evolution from compensated hypertrophy to HF by diminishing MC degranulation in mice after SAC-induced pressure overload (Hara et al., 2002). Together, these preclinical studies suggest that MC stabilizers are potential therapeutic agents for pressure overload-induced hypertrophy and HF, although clinical studies are warranted to validate their efficacies.

## Monocytes and Macrophages

**FIGURE 3:**
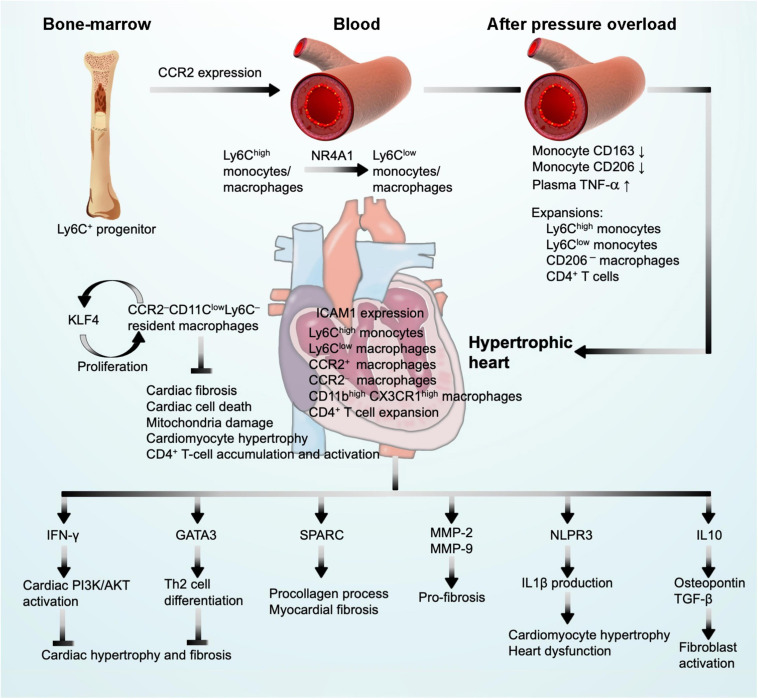
Monocyte and macrophage function in pressure overload-induced cardiac hypertrophy and fibrosis. CCR2 expression drives Ly6C^+^ monocyte progenitor trafficking to peripheral blood where monocyte differentiation may occur. After pressure overload injury, blood monocytes undergo phenotypic alterations and monocyte/macrophage expansion. These cells infiltrate into the heart where they produce IFN-γ and GATA3 to block cardiac hypertrophy and fibrosis, produce SPARC, MMP-2, MMP-9, IL1β, and IL10 to promote cardiac hypertrophy and cardiac fibrosis, leading to cardiac dysfunction, and express KLF4 to promote macrophage self-proliferation.

Monocytes contain heterogeneous subsets that can be divided by their surface expression of Ly6C and chemokine C-C motif receptor-2 (CCR2), including classical Ly6C^high^CCR2^high^ monocytes and non-classical Ly6C^low^CCR2^low^ monocytes (Geissmann et al., 2003). Ly6C^high^ monocytes are derived from Ly6C^+^ progenitors in the bone marrow. Relying on the expression of CCR2, Ly6C^high^ monocytes are released into the blood (Serbina and Pamer, 2006). Ly6C^high^ monocytes give rise to Ly6C^low^ monocytes through a nuclear receptor subfamily-4-dependent (NR4A1) transcriptional program (Hanna et al., 2011). It is commonly thought that macrophages merely arise from circulating blood monocytes. With the advent of fate mapping, parabiosis, and adoptive transfer techniques, studies demonstrated that tissue-resident macrophages replenish themselves mainly by local proliferation in steady-state heart (Ginhoux et al., 2010; Schulz et al., 2012; Hashimoto et al., 2013; Yona et al., 2013). These resident macrophages originate from embryonic yolk-sac progenitors independent of bone-marrow-derived monocytes (Epelman et al., 2014) (Figure 3). In contrast, during the perturbed state caused by hemodynamic stress, such as pressure overload and even ischemic injuries, the majority of macrophages are recruited and differentiated from blood monocytes (Molawi et al., 2014). Reacting to the marked upregulation of chemokines, mainly CCL2, CCL7, CCL12, and monocyte-chemoattractant protein (MCP-1) (Dewald et al., 2005; Hashimoto et al., 2013; Hilgendorf et al., 2014; Patel et al., 2018) and chemokine receptors CCR1, CCR2, CCR5, and C-X3-C motif chemokine receptor-1 (CX3CR1) (Weisheit et al., 2014, 2021; Nemska et al., 2016), Ly6C^high^CCR2^high^ and Ly6C^low^CX3CR1^high^ monocytes and macrophages infiltrate into hypertrophic hearts using CCR2 and CX3CR1 within the first week after pressure overload injury (Weisheit et al., 2014, 2021; Nemska et al., 2016; Patel et al., 2017, 2018; Liao et al., 2018; Figure 3).

### Monocyte and Macrophage Functions in Pressure Overload-Induced Cardiac Hypertrophy and Fibrosis

Cardiac monocytes and macrophages exert significant effects in response to pressure overload-induced adverse cardiac hypertrophy and fibrosis. A series of studies illustrated that SAC surgery in rats induced myocardium macrophage infiltration and fibroblast activation at the early phase of hypertrophy (Kuwahara et al., 2003, 2004). TAC-induced cardiac hypertrophy exhibited expansion of circulating Ly6C^high^ and Ly6C^low^ monocytes and pro-inflammatory CD206^–^ cardiac macrophages at 1 week after surgery, prior to significant cardiac hypertrophy and dysfunction (Weisheit et al., 2014, 2021; Nemska et al., 2016; Patel et al., 2017, 2018; Liao et al., 2018; Figure 1B). Recent studies showed that peripheral monocytes from hypertension patients with or without LV hypertrophy also underwent phenotypic alterations. Patients with hypertension but without LV hypertrophy showed reduced monocyte surface CD163 expression and plasma IL10 levels, but increased plasma TNF-α. LV hypertrophy further reduced monocyte surface expression of CD163 and CD206 and increased plasma TNF-α. Treatment with antihypertensive drug irbesartan increased monocyte CD163 and CD206 expression in LV hypertrophic patients (Zhang et al., 2021; Figure 3), although it remains untested whether the similar alterations occurred in monocytes within the hypertrophic heart.

In TAC-induced hypertrophic mice, while neutrophils peaked at 3 days after injury, Ly6C^low^ and Ly6C^high^ macrophages peaked at 6 days after TAC (Figure 1A, bottom panel, and Figure 1B, upper and bottom panels). These macrophages expressed high levels of surface CD11b and CX3CR1. At this time point, myocardium expressed high levels of adhesion molecule intercellular adhesion molecule 1 (ICAM-1) on cardiac endothelial cells. Use of fluorescence microscopy detected Ly6C^low^ macrophage attachment on the intra- and extravascular vessel wall (Weisheit et al., 2014). These observations may explain the Ly6C^low^ macrophage accumulation in cardiac tissue after TAC. A recent study further tested the role of macrophages in the late phase of cardiac hypertrophy and fibrosis. Pressure overload-induced hypertrophy generated a two-phase response of cardiac macrophages. During the early compensated phase (1 week), there was an outburst of local infiltration and proliferation of macrophages. During the late decompensated phase (4 weeks), the infiltration of Ly6C^high^CX3CR1^+^CCR2^+^ classic monocytes increased. Macrophages displayed a second phase of proliferation (Figure 1B, bottom panel). Macrophages expressed Kruppel-like factor 4 (KLF4), a key transcription factor that regulates macrophage proliferation and angiogenesis (Liao et al., 2018; Figure 3). Resident macrophages are involved in adaptive response to pressure overload. Deficiency of KLF4 reduced resident macrophage proliferation and increased cardiac tissue fibrosis, cell death, mitochondria damage, and cardiomyocyte hypertrophy (Liao et al., 2018). Clodronate (CL) liposomes have been used to deplete cardiac macrophages. CL binds to the intracellular ATP and inhibits ATP function, leading to macrophage apoptosis (Frith et al., 1997). Liposomes selectively deliver CL into macrophages *via* their phagocytic activity. Repeated administration of CL may also deplete macrophages and monocytes from the bone marrow and in peripheral blood (Robbins et al., 2013). In HF-prone hypertensive Ren-2 rats that carry two copies of the mouse renin-2 gene and exhibit moderate hypertension, myocardial hypertrophy, and excessive fibrosis likely due to Ang-II overproduction, intravenous administration of liposomal CL selectively depleted blood monocytes and myocardium macrophages. Macrophage depletion increased cardiac cell apoptosis and myocardial CD4^+^ T-cell accumulation (Figure 3), thereby worsening the rat cardiac function with reduced EF, fractional shortening (FS), and heart weight/bodyweight (HW/BW) ratio, and increased end diastolic volume at 6 weeks after macrophage depletion. Therefore, macrophage depletion associated with myocardial dysfunction in hypertensive rats (Zandbergen et al., 2009; Figure 3). Yet, opposite observations were also made. In salt-sensitive Sabra rats with high-salt diet-induced cardiac hypertrophic mice, macrophage depletion with CL liposomes for 4 weeks blocked the elevation of systolic blood pressure, reduced cardiac fibrosis and hypertrophy, and protected cardiac function (Kain et al., 2016). Similar observations were made from the same rats that underwent TAC-induced cardiac hypertrophy. At 3 weeks after surgery with and without macrophage depletion, CL treatment also protected mice from LV hypertrophy with significantly reduced cardiac fibrosis and expression of hypertrophic genes, including TGF-β, collagen type IIIα-1, and atrial natriuretic factor (ANF) (Kain et al., 2016). These conflicting results remain unexplained. It is probably due to the differences in rat strains. Under the same treatments of high-salt diet or TAC-induced cardiac hypertrophy, CL-mediated macrophage depletion in Sprague Dawley rats did not affect myocardial T-cell accumulation, cardiac cell death, or cardiac function, although these results were not explained (Zandbergen et al., 2009).

### Molecular and Cellular Mechanisms of Monocyte and Macrophage Actions

Monocytes and macrophages regulate cardiac fibrosis by clearing dead cells and debris, secreting growth factors and cytokines, controlling fibroblast activation, and producing proteases for ECM degradation (Kagitani et al., 2004; Wynn and Barron, 2010; Ren et al., 2011). Macrophages and monocytes are rich sources for pro-inflammatory cytokines IL1β and TNF-α; anti-inflammatory cytokines and growth factors IL10, PDGF, and TGF-β; and proteases MMP2 and many other MMPs (Fadok et al., 1998; Huynh et al., 2002). These macrophage- and monocyte-derived cytokines, growth factors, and proteases are released during the process of cardiomyocyte hypertrophy and fibroblast activation to promote cardiac cell death and initiate the ingestion of apoptotic cardiomyocytes and necrotic debris.

Cardiac macrophages contain steady-state CCR2^–^CD11c^low^Ly6C^–^ resident macrophages that proliferate *in situ* under physiological conditions (Liao et al., 2018) (Figure 1B, bottom panel, and Figure 3). Under inflammatory conditions, Ly6C^high^ monocytes infiltrate into the myocardium and differentiate into CCR2^+^ and CCR2^–^ mature macrophages that account for the majority of cardiac macrophages to coordinate cardiac inflammation (Epelman et al., 2014; Figure 3). Several studies have reported that the secretory molecules from macrophages contributed to the development of cardiac hypertrophy and fibrosis. It is the CCR2^+^ macrophages that produce IL1β *via* the NLPR3 inflammasome mechanism under cardiac stress as a mechanism to promote cardiomyocyte hypertrophy and contractile dysfunction (Palmer et al., 1995; Harada et al., 1999; Figure 3). Therefore, blocking monocyte cardiac influx and consequent CCR2^+^ macrophage expansion was cardioprotective, whereas complete depletion of macrophages that also target resident CCR2^–^ macrophages abolished macrophage cardioprotective function (Kaikita et al., 2004; van Amerongen et al., 2007). Macrophages are the main source of MMPs, including MMP-1, -7, -8, -9, -12, and -13, among which MMP-2 might be the most relevant pro-fibrotic MMP (Heymans et al., 2005; Lim et al., 2006; Murray and Wynn, 2011; Matsusaka et al., 2016). Deficiency of MMP-2 reduced TAC-induced anterior and posterior wall thickness, LV mass, LV systolic and diastolic blood pressures, LV weight/BW ratio, cardiomyocyte dilation, and cardiac fibrosis (Matsusaka et al., 2016). Under a similar condition, MMP-9 deficiency showed moderate effect on TAC-induced hypertrophy in mice (Heymans et al., 2005; Figure 3). Macrophages can also resolve fibrosis in the process of reparative phase by expressing high levels of TIMPs and MMP-13 (D’Angelo et al., 2001). Salty drinking water unilateral nephrectomy aldosterone (SAUNA) infusion-induced hypertension or natural aging for 18 or 30 months increased heart macrophage and Ly6C^high^ monocyte accumulation. CCR2 deficiency significantly blunted such accumulations. These cardiac macrophages express IL10. Selective depletion of IL10 in cardiac macrophages in *Cx3cr1 Il10^–/–^*mice improved SAUNA-induced cardiac fibroblast activation, collagen deposition, and LV diastolic dysfunction. A mechanistic study suggested that IL10 controls cardiac macrophage expression of osteopontin and TGF-β, thereby inducing cardiac fibroblast expression of fibrotic proteins collagen and fibronectin (Hulsmans et al., 2018; Figure 3). Macrophage-derived SPARC (secreted protein acidic and rich in cysteine) is an ECM-associated protein that affects cardiac collagen disposition and cardiac stiffness (Figure 3). Macrophages represent a source of increased myocardium SPARC in a model of pressure overload-induced cardiac fibrosis. SPARC production in the myocardium followed a time course after pressure overload induction. SPARC production was not significantly changed at the beginning of pressure overload induction (3 days), but increased at 1 and 4 weeks following pressure overload. This expression pattern coincided with myocardium accumulation F4/80-positive macrophages as detected by immunohistochemistry and flow cytometry (McDonald et al., 2018; Figure 1B, upper panel).

In contrast to aforementioned pro-hypertrophic molecules, IFN-γ is a common pro-inflammatory but anti-hypertrophic cytokine expressed in CD68^+^ macrophages. IFN-γ-deficient mice subjected to TAC resulted in a remarkable maladaptation of hypertrophy and fibrosis. Mechanistic studies showed that the cardiac activation of the PI3K/Akt signaling pathways is a key signaling pathway in IFN-γ-controlled compensatory hypertrophy (Kimura et al., 2018; Figure 3). GATA3 acts as a zinc-finger transcription factor and mediates Th2 cell differentiation (Figure 3). In T cells, GATA3 is specifically induced by IL4 through activation of its proximal promoter (Scheinman and Avni, 2009). Recent studies showed that GATA3 also played important roles in monocyte and macrophage pathobiology during cardiac remodeling (Yang et al., 2018). In myeloid-specific GATA3-deficient mice that were generated by crossing GATA3 floxed (*GATA3*^*fl/fl*^) mice with LysM^Cre^ mice, TAC-induced cardiac dysfunction and adverse LV remodeling were much improved compared with those in the *LysM*^*Cre*^ control mice. A large number of pro-inflammatory Ly6C^high^ monocytes and macrophages and fewer reparative Ly6C^low^ macrophages are located in the myocardium of *LysM*^*Cre*^ control mice (Yang et al., 2018).

In pressure overload-induced hypertrophic mice, recruitment of Ly6C^high^CCR2^+^ monocytes caused significant expansion of cardiac CD3^+^CD8^+^ and CD3^+^CD4^+^ T cells. Anti-CCR2 antibody treatment did not affect such expansion in the myocardium, although this antibody blocked the expansion of these T cells in the heart draining lymph nodes (Patel et al., 2018), suggesting that cardiac T-cell expansion does not involve CCR2. Earlier studies showed that pressure overload activated cardiac T cells. The kinetics of cardiac T-cell infiltration associated with systolic dysfunction (Nevers et al., 2015). Using lymphocyte deficient RAG2-deficeint mice, T-cell-deficient *TCRα^–/–^* mice, CD4^+^ T-cell-selective depleted mice, CD8^+^ T-cell-selective depleted mice, and T-cell co-stimulation blocker abatacept, studies showed that cardiac CD4^+^ T cells contribute to cardiac hypertrophy by promoting cardiac tissue fibrosis and inflammation (Laroumanie et al., 2014; Nevers et al., 2015; Kallikourdis et al., 2017). These results present additional mechanisms of cardiac monocyte infiltrates in pressure overload-induced hypertrophy (Figure 3).

### Therapies by Targeting Monocytes and Macrophages

Therapeutic interventions targeting monocytes and macrophages by selectively depleting cardiac monocytes and macrophages or blocking the infiltration of circulating monocytes and macrophages might be promising approaches to alleviate pressure overload-induced cardiac hypertrophy and fibrosis. Targeting macrophage adhesion molecules or chemokine receptors that mediate macrophage adhesion and migration tested these possibilities. ICAM-1 is implicated in macrophage recruitment. In SAC-induced pressure overload and cardiac hypertrophy in rats, ICAM-1 was expressed in the intramyocardial coronary arteries at 1 day after surgery and peaked in 3 days. Immunohistochemistry indicated that CD68^+^ macrophages were clustered next to these arteries. Antibody-mediated neutralization of ICAM-1 blocked myocardial macrophage accumulation and reduced fibroblast proliferation, TGF-β1 expression, and myocardial fibrosis, although arterial pressure and LV or cardiomyocyte hypertrophy did not differ from those treated with control IgG (Kuwahara et al., 2003).

Targeting monocyte/macrophage infiltration has also been proven effective to mitigate cardiac damage after pressure overload-induced hypertrophy. In chemokine Fraktalkine receptor CX3CR1-deficient *Cx3cr1*^*GFP/GFP*^ mice, TAC-induced reductions in EF and cardiac output were fully recovered, along with reduced HW/BW, cardiac damage marker aldolase, cardiac hypertrophy and its marker B-type naturetic peptide (BNP), and cardiac fibrosis (Weisheit et al., 2021). Deficiency of CCR2 blocked cardiac tissue macrophage infiltration, increased myocardium capillary density, and improved cardiac function, although it did not affect cardiac tissue fibrosis and cardiomyocyte hypertrophy (Liao et al., 2018). These observations support a detrimental role of Ly6C^high^CX3CR1^+^CCR2^+^ classic monocytes and macrophage in exacerbating TAC-induced cardiac hypertrophy. Interestingly, global ablation of macrophages together with DCs with AP20187 starting at 2 weeks after TAC surgery showed moderate effect in cardiac function and fibrosis (Patel et al., 2017). These negative results suggest that cardiac macrophages play a different role at different time courses after cardiac injury. Cardiac macrophage depletion before macrophages peak at 1 week after TAC surgery may yield different results. It is also possible that different types of macrophages act differently in pressure overload-induced hypertrophy. In TAC-treated mice, immediate depletion of cardiac macrophages with CL reduced anterior wall thickness, LV volume and mass, cardiomyocyte thickness, blood wall thickness, and LV fibrosis (Kain et al., 2016). In the same model, intraperitoneal injection of CCR2 antagonist RS504393 starting at day 3 after TAC surgery for 4 days blocked cardiac macrophage accumulation, reduced cardiac vascular cell adhesion protein 1 (VCAM-1) expression, reduced HW/TL and cardiac fibrosis, and improved cardiac hypertrophy and cardiac function. Similar observations were made when the mice were treated with anti-CCR2 antibody (Patel et al., 2018). Together, results from these studies suggest that it is only effective to minimize pressure overload-induced cardiac injury by targeting monocyte/macrophage chemotaxis before or immediately after the pressure overload injury.

## Neutrophils

Neutrophils are the first leukocytes that appear in the myocardium following pressure overload-induced hypertrophy, within 3 days after the injury (Weisheit et al., 2014, 2021) (Figure 1A, bottom panel). In patients with concentric or eccentric LV hypertrophy, blood neutrophil-to-lymphocyte ratio (NLR) was elevated and correlated strongly with the LV mass index (*r* = 0.508, *p* < 0.001) (Afşin et al., 2019). Neutrophil transmigration is dependent on endothelial cell activation and subsequent expression of adhesion molecules (Woodfin et al., 2009; Filippi, 2019), a process that is enhanced by inflammatory stimuli, such as TNF-α, IL1β, and even MC-derived histamine (Mackay et al., 1993; Asako et al., 1994; Sahni et al., 2005). In an autocrine fashion, neutrophil activation stimulates cardiac fibroblast release of IL6 to upregulate endothelial cell ICAM-1 expression as a mechanism to attract further neutrophils and macrophages (Hofbauer et al., 2019).

### Neutrophil Function in Pressure Overload-Induced Cardiac Hypertrophy and Fibrosis

In response to pressure overload, alterations in myocardium neutrophil contents associate with cardiac hypertrophy and fibrosis. In a mouse inter-renal aortic banding-induced LV hypertrophy model, histopathological and immunohistochemical examinations revealed that macrophage and neutrophil infiltration in LV and RV appeared next to the coronary arteries, containing abundant ICAM-1 immunostaining signals in the first 3 days after injury, long before vascular wall thickening, perivascular fibrosis (10 days), and cardiomyocyte hypertrophy (28 days). Cardiac neutrophils remained high until 42 days after aortic banding (Higashiyama et al., 2007). Similar observations were made in mice after TAC-induced cardiac hypertrophy. Cardiac neutrophils peaked in 3 days after TAC surgery, followed by macrophages in 6 days as determined by flow cytometry and fluorescence microscopy. Like those in inter-renal aortic banding-induced LV hypertrophic mice, cardiac neutrophil contents remained high from 3 to 21 days after TAC surgery, and possibly much longer (Weisheit et al., 2014; Figure 1A, bottom panel).

Myocardium neutrophils in hypertrophic heart play detrimental roles. In TAC-injured hypertrophic mice, neutrophil depletion with injection QOD of anti-mouse Ly6G antibody starting from 2 days before surgery for 2 weeks significantly reduced HW/TL ratio, posterior wall thickness, LV systolic and diastolic diameters, and cardiomyocyte hypertrophy, and increased EF. In 2 days after TAC, FACS analysis showed that neutrophil depletion blocked cardiac monocyte and macrophage accumulation (Wang et al., 2019b). Wnt signaling regulates cell proliferation, differentiation, polarity, adhesion, and motility (van Amerongen and Nusse, 2009; Wend et al., 2010). Wnt5a is a noncanonical Wnt that stimulates neutrophil chemotactic migration (Jung et al., 2013). Depletion of Wnt5a in myeloid cells in *Wnt5a^*f/f*^LysM^*Cre/*+^* mice significantly blocked cardiac inflammatory cell (neutrophils, Ly6C^*hi*^ monocytes, and macrophages) infiltration and myocardium pro-inflammatory cytokine and chemokine (IL1β, IL6, CXCL1, CXCL2, CXCL5, and CCL2) expression, and repaired TAC-induced cardiac dysfunction with reduced cardiomyocyte hypertrophy and cardiac fibrosis at 1, 4, and 8 weeks after TAC injury. In contrast, LysM-Cre-mediated myeloid cell overexpression of Wnt5a enhanced TAC-induced myocardium neutrophil accumulation and pro-inflammatory cytokine and chemokine expression and worsened TAC-induced cardiac dysfunction, cardiac cell hypertrophy, and fibrosis (Wang et al., 2019b). Yet, the limitation of this study is that the use of *LysM^*Cre/*+^* mice is not selective to neutrophils, but rather all myeloid cells. A better model is required.

**FIGURE 4:**
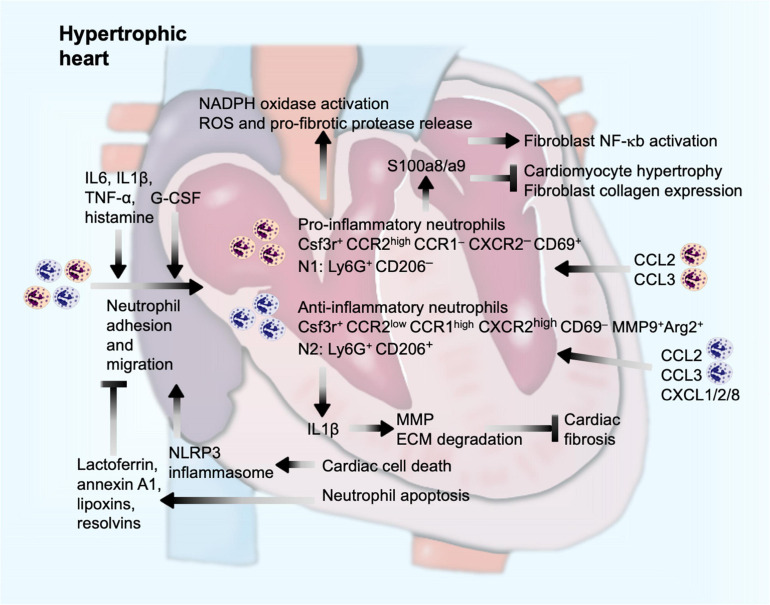
Neutrophil function in pressure overload-induced cardiac hypertrophy and fibrosis. Cardiac injury and inflammatory molecules promote neutrophil adhesion and migration into the heart, a process that can be enhanced by G-CSF and neutrophil-derived Wnt5a, but blocked by lactoferrin and anti-inflammatory lipid-mediators from apoptotic cardiac cells or neutrophils. Inside the heart, neutrophils produce IL1β to promote ECM proteolysis and reduce cardiac fibrosis, induce activation of NADPH oxidase and release of ROS and pro-fibrotic proteases, or produce S100a8/a9 to induce fibroblast inflammatory activation or to block cardiomyocyte hypertrophy or fibroblast procollagen expression.

The modern technique allowed detailed analysis of myocardium inflammatory infiltrates after pressure overload-induced cardiac hypertrophy. Single-cell RNA sequencing analysis of cardiac CD45^+^ cells revealed 20 clusters of immune cells using the two-dimensional t-distributed stochastic neighbor embedding visualization. This technique demonstrated the presence of two distinct neutrophil clusters in the myocardium from sham-operated or TAC-treated mice. Although their functional differences in hypertrophic heart were not compared, both clusters of neutrophils showed different gene expression profiles. Both clusters expressed the neutrophil marker Csf3r and got expanded at 4 weeks after TAC injury. One cluster of neutrophils expressed high levels of chemokine receptor Ccr2 but not Ccr1 and Cxcr2. These cells also expressed the cell activation marker CD69. Unsupervised gene signature analysis revealed significant correlation of this cluster of neutrophils with transcription activity and antigen presentation signatures. In contrast, the other cluster of neutrophils expressed high levels of Ccr1 and Cxcr2 but low levels of Ccr2. These cells did not express CD69, but anti-fibrotic MMP-9 and arginase-2, essential for IL10-mediated anti-inflammatory responses (Dowling et al., 2021). Therefore, these two populations of neutrophils represent pro-inflammatory and anti-inflammatory activities in hypertrophic hearts and expand parallelly after pressure overload injury (Martini et al., 2019; Figure 4), similar to the Ly6C^*high*^ and Ly6C^*low*^ pairs of monocytes/macrophages. Such unique expression profile of cell surface chemokine receptors on these two neutrophil populations suggests that these neutrophils use different sets of chemokines for their migration and cardiac accumulation. CCR2^+^ pro-inflammatory neutrophils may predominantly use CCL (e.g., CCL2 and CCL3) as their chemokines, whereas CCR1^+^CXCR2^+^ anti-inflammatory neutrophils may use both CCL (e.g., CCL2 and CCL3) and CXCL (e.g., CXCL1, CXCL2, and CXCL8) chemokines to mediate their chemotactic migration (Capucetti et al., 2020; Figure 4).

Pressure overload-induced hypertrophy and consequent debanding (pressure unload) mimic human aortic stenosis and later aortic valve replacement. Different from those of only pressure overload-induced cardiac hypertrophy, neutrophils played a cardioprotective role in pressure unloaded hypertrophic mice when mice were treated with granulocyte colony-stimulating factor (G-CSF). G-CSF is a hematopoietic cytokine that regulates the proliferation, differentiation, and survival of myeloid progenitor cells and plays an eminent role in the regulation and production of neutrophil granulocytes. G-CSF treatment significantly reversed pressure overload-induced cardiac dysfunction and remodeling accompanied by a selective release of IL1β. Regression of cardiac hypertrophy by G-CSF generated a considerable myocardium neutrophil infiltration. A mechanistic study suggested that G-CSF-induced neutrophil infiltration increased myocardium IL1β expression that induced cardiac fibroblast expression of collagenases MMP-2 and MMP-9 or other ECM-degrading proteases, thereby assisting the regression of cardiac fibrosis (Szardien et al., 2012; Figure 4).

### Mechanisms of Neutrophil Action

In response to the acute inflammation, neutrophils are recruited to eliminate dead cells and matrix debris under normal reparative conditions (Bratton and Henson, 2011; Sreejit et al., 2020). However, excessive accumulation of neutrophils or delayed clearance of debris accelerates cardiomyocyte injury, which leads to further fibrotic process (Bratton and Henson, 2011). Similar to the two clusters of neutrophils described in mouse hypertrophic hearts (Martini et al., 2019), earlier studies reported pro-inflammatory N1 neutrophils and anti-inflammatory N2 neutrophils from mouse infarcted hearts (Ma et al., 2016). While N1 neutrophils express pro-inflammatory CCL3, CCL5, IL1β, IL6, IL12, and TNF-α at the early phase of heart infarction, N2 express anti-inflammatory CD206, IL10, TGF-β, arginase-1, and Ym1 at the late phase of heart infarction. FACS, immunofluorescent double staining, and RT-PCR confirmed that N1 neutrophils were Ly6G^+^CD206^–^ that can be induced by LPS together with IFN-γ. N2 neutrophils were Ly6G^+^CD206^+^ that can be induced by IL4 (Ma et al., 2016), similar to M1 and M2 macrophages that we reported previously (Zhou et al., 2015). Therefore, the roles of N1 and N2 neutrophils in hypertrophic hearts may mimic those of M1 and M2 macrophages or Ly6C^high^ and Ly6C^low^ monocytes, although limited information is available to support this comparison.

N1 neutrophils degranulate and release MMPs, elastase, proteoglycans, and cathepsin G that mediate collagenous and non-collagenous connective tissue catabolism (Ma et al., 2013). In response to pressure overload injury, neutrophilic nicotinamide adenine dinucleotide phosphate (NADPH) oxidase gets activated (Li et al., 2002), leading to neutrophil degranulation and release of pro-fibrotic proteases and reactive oxygen species (ROS) (Ciz et al., 2012). Necrotic cell death after pressure overload (Nadal-Ginard et al., 2003) release adenosine triphosphate to activate NLRP3 inflammasomes as a mechanism to develop an inflammatory microenvironment for neutrophils to adhere and infiltrate (McDonald et al., 2010; Figure 4). In contrast, apoptotic cells can also release lactoferrin, a pleiotropic glycoprotein with anti-inflammatory properties and annexin A1 to block neutrophil adhesion and migration, thereby minimizing cardiac damage from pressure overload (Bournazou et al., 2009; Qin et al., 2019). Similarly, apoptotic neutrophils or tissue macrophages also release anti-inflammatory lipid mediators (lipoxins and resolvins) to increase apoptotic neutrophil CCR5 expression to remove neutrophil chemokine CCL3 and CCL5 as a mechanism to block consequent neutrophil migration and recruitment (Soehnlein and Lindbom, 2010; Figure 4).

The time course of cardiac neutrophil infiltration may impact cardiac function and remodeling differently after hypertrophic injury. Although there is currently no direct evidence, this hypothesis has been tested in other cardiac injury models. During the acute phase of eccentric hypertrophy in rats induced by aortocaval fistula (ACF), anti-rat neutrophil antibody-induced neutrophil depletion in LV myocardium 2 days before the surgery prevented myocardium MMP activation, collagen loss, and cardiomyocyte apoptosis at the first 24 h after the surgery and attenuated the development of eccentric hypertrophy at 2 and 3 weeks. These observations suggest that neutrophils enhance the progress of ACF-induced rat cardiac hypertrophy. In contrast, sustained neutrophil depletion over 4 weeks resulted in adverse cardiac remodeling with further increases in cardiac dilatation and macrophage infiltration (Kolpakov et al., 2009). In mouse infarcted hearts, pro-inflammatory Ly6G^+^ N1 neutrophils accumulated at day 1 after myocardial infarction (MI) and then sharply reduced to the baseline at 7 days after infarction. In contrast, anti-inflammatory Ly6G^+^CD206^+^ N2 neutrophils started accumulating at 5 days after infarction and peaked at 7 days after infarction (Ma et al., 2016). The neutrophil populations at the early and late phases of cardiac hypertrophy may act differently. Chronic anti-neutrophil therapy against cardiac remodeling might not achieve the expected benefits.

Similar complications occurred at the molecular levels. Neutrophils produce S100a8/a9 that acts as an initial pro-inflammatory factor to trigger cardiac inflammation and fibrosis after pressure overload injury. Immunofluorescent staining revealed S100a8/a9 expression primarily from infiltrated neutrophils in mouse heart at 1 day after Ang-II infusion. Mouse cardiomyocytes and cardiac fibroblasts all express the S100a8/a9 receptors, including RAGE product and TLR4, independent of Ang-II stimulation (Wu et al., 2014). Therefore, neutrophil-derived S100a8/a9 may act on both major cardiac cells. To cardiac fibroblasts, S100a8/a9 induced the expression of a panel of cytokines and chemokines by activating the inflammatory nuclear factor-κB (NF-κB) pathway. In mice, S100a8/a9 neutralization with its antibody blocked Ang-II infusion-induced NF-κB activation, inflammatory cell (CD45^+^, CD11b^+^, CD45^+^CD11b^+^, and Gr1^+^ cells) infiltration, and cytokine (IL1β and TNF-α) and chemokine (CCL2, CCL3, CCL5, and CCL7) production, in addition to reduced cardiac interstitial fibrosis and cardiac cell hypertrophy (Wu et al., 2014; Figure 4). In contrast, S100a8/a9 plays a protective role against rat cardiomyocyte hypertrophy and cardiac fibroblast collagen expression. To cultured rat neonatal ventricular cardiomyocytes, norepinephrine induced cardiomyocyte hypertrophy, including elevated expression of atrial natriuretic peptide (ANP) and β-myosin heavy chain. Use of S100a8/a9 significantly blocked these activities of norepinephrine. S100a8/a9 RNA silencing exacerbated norepinephrine-induced rat neonatal cardiomyocyte hypertrophy (Wei et al., 2015; Figure 4). The same study also showed cardioprotective activity of S100a8/a9 against rat cardiac fibroblasts. Use of S100a8, S100a9, or S100a8/a9 significantly blocked the expression of pro-collagen I and III from cultured rat cardiac fibroblasts (Wei et al., 2015). It remains unclear why this study yielded opposite conclusion from those of Wu et al. (2014) or those from most other studies (Volz et al., 2012) besides the fact that this study used rat cells. Together, the role of neutrophils in cardiac hypertrophy remains elusive.

## Dendritic Cells

Dendritic cells are professional antigen-presenting cells that are capable of sensing chemoattracting inflammatory signals to mobilize and migrate to the regions of tissue injury, where DCs phagocytose dead cells and matrix debris as macrophages do in addition to their professional function to activate T cells (Banchereau et al., 2000). Common DC progenitors are heterogeneous, including those IFN-α-producing pDCs and cDCs (often called myeloid DCs) (Manh et al., 2013). CCR1 and CCL3 mediate the homing of immature DCs, while CCR7 and CXC-chemokine receptor type 4 (CXCR4) regulate mature DC homing to regional lymph nodes (Sallusto et al., 1999; Randolph et al., 2008; Delgado-Martín et al., 2011). Mature DCs travel to secondary lymphoid tissue to deliver antigenic peptides to T cells. T cells are stimulated by antigenic peptides bound to the major histocompatibility complex (MHC) molecules on DCs and then release IL12, IL23, and IL27 after activation (Curtsinger and Mescher, 2010; Kimura et al., 2016; Li et al., 2019). T-cell activation results in T-cell proliferation and differentiation into their subtypes, such as regulatory T cells (Tregs), T helper cells (Th), and killer T cells.

### DCs in Pressure Overload-Induced Cardiac Hypertrophy and Fibrosis

In response to pressure overload, bone-marrow-derived CD11c^+^ DCs promote cardiac hypertrophy and fibrosis. In TAC-induced cardiac hypertrophic mice, the number of CD11c^+^ cells and the percentage of CD11c^+^MHC-II^+^ (major histocompatibility complex class II molecule positive) DCs were increased in the LV myocardium, spleen, and peripheral blood. Diphtheria toxin (DT)-induced depletion of CD11c^+^ DCs in irradiated WT recipient mice that received bone marrow transfer from CD11c-DTR/GFP transgenic donor mice significantly blocked TAC-induced cardiac dysfunction along with reduced cardiomyocyte hypertrophy, cardiac fibrosis, LV remodeling, and LV myocardium CD45^+^ cells, CD11b^+^ cells, CD8^+^ T cells, or activated effector CD44^+^CD8^+^ T cells at 24 weeks after TAC injury. LV tissue homogenate from hypertrophic mice promoted DC activities in activating CD4^+^ and CD8^+^ T cells. These observations suggest that bone-marrow-derived CD11c^+^ DCs play a maladaptive role in hemodynamic overload-induced cardiac inflammation, hypertrophy, and fibrosis through the presentation of cardiac self-antigens to T cells (Wang et al., 2017).

Time course study tested DC expansion in different organs after TAC-induced hypertrophy in mice. CD11c^+^MHC-II^+^ cDCs accumulated in the heart tissue in a biphasic manner, with peaks at both early (1 week) and late (8 weeks) phases. In contrast, CD11c^low^MHC-II^+^B220^+^ pDCs peaked at 1 week after TAC surgery (Figure 1A, bottom panel). Although studies did not test whether cDCs and pDCs acted differently in pressure overload-induced hypertrophy, global ablation of DCs together with macrophages with AP20187 at 2 weeks after TAC injury did not affect hypertrophy. AP20187 dimerizes the cytoplasmic Fas fragments to induce Fas-induced mononuclear phagocyte apoptosis (Burnett et al., 2004). Yet, results from this study did not specify the role of cDCs or pDCs and were confounded by non-selective ablation of macrophages and monocytes. Furthermore, DC ablation was performed after the heart DCs passed the first week peak time after TAC surgery (Patel et al., 2017).

In CD11c^+^DOG mice, DT-induced depletion of DCs reduced aldosterone and high-salt diet-induced cardiac hypertrophy, perivascular fibrosis, expression of cardiac collagen, connective tissue growth factor, lipocalin, and hypertrophic marker BNP (Araos et al., 2019). CD11c^+^DOG mice are transgenic mice in which the DT receptor gene is expressed under the control of the CD11c promoter (Hochweller et al., 2008). In humans, blood cDC and pDC contents increased in HF patients with NYHA (New York Heart Association) class II with coronary artery disease (CAD) and further increased in patients with NYHA class III–IV, although such increases did not reach statistical significance. Blood cDC, pDC, and total DC counts were highest in NYHA III–IV patients with non-ischemic dilated cardiomyopathy. Yet, blood cDC and pDC counts did not associate with LV EF or systolic or diastolic functions (Athanassopoulos et al., 2009). Together, studies from pressure overload-induced hypertrophy models and human studies support a pathogenic role for DCs in this cardiac disease.

### Mechanisms of DC Function

**FIGURE 5:**
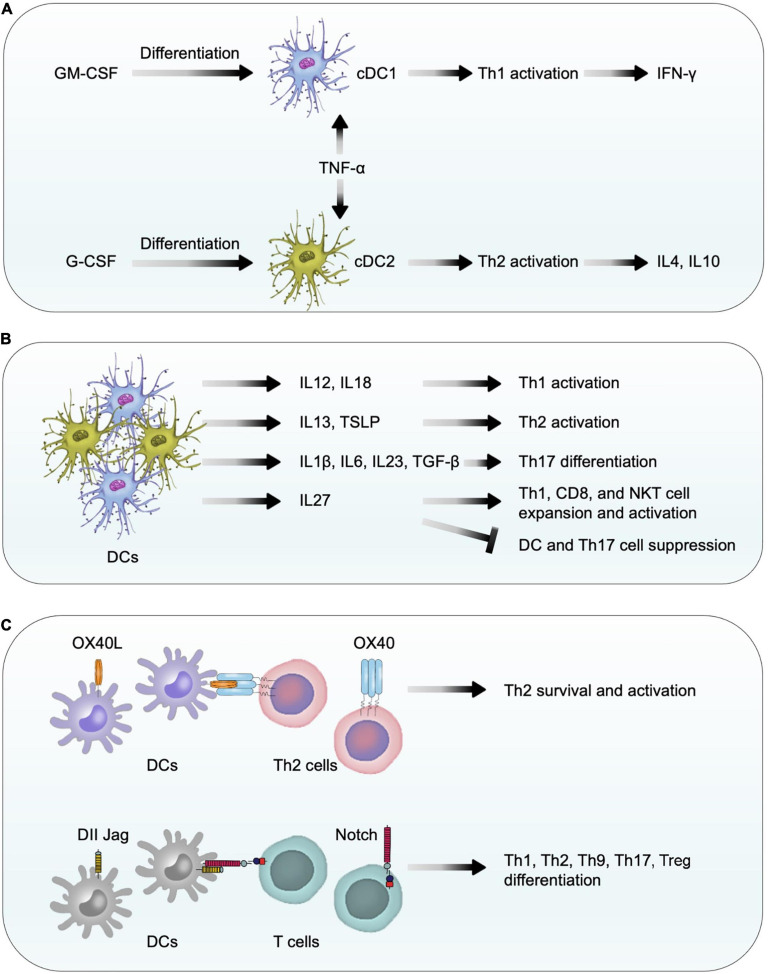
Dendritic cell function in pressure overload-induced cardiac hypertrophy and fibrosis. **(A)** In response to GM-CSF and G-CSF, bone-marrow-derived CD11c^+^ DCs differentiate into conventional DC1 and DC2 cells. TNF-α activates conventional DC1 and DC2 to release Th1 and Th2 cytokines to affect cardiac fibroblast activation, fibrosis, and cell death. **(B)** DCs release different types of cytokines to control T-cell differentiation, expansion, and activation. **(C)** Using co-stimulatory molecules OX40L, Delta-like ligands, and Jagged, DCs promote the survival, differentiation, and activation of different T-cell subtypes.

While the pleiotropic myelopoietic growth factor granulocyte-macrophage colony-stimulating factor (GM-CSF) (Zhan et al., 2019) induces immature DC differentiation into myeloid DCs (also called conventional DC1) (Greter et al., 2012; Becher et al., 2016), G-CSF induces conventional DC2 differentiation (Arpinati et al., 2000; Shaughnessy et al., 2006). TNF-α-activated DC1 stimulate Th1 cell activation and release of IFN-γ, whereas TNF-α-treated DC2 activate Th2 cells to release IL4 and IL10 (Arpinati et al., 2000; Figure 5A). DCs release different cytokines and express different co-stimulatory molecules to activate different T-cell subsets. For example, DCs release IL12 and IL18 to facilitate Th1 differentiation (Bellinghausen et al., 2003) but release IL13 and TSLP (thymic stromal lymphopoietin) to promote Th2 cell priming and expansion (Bellinghausen et al., 2003; Perrigoue et al., 2009; Okoye and Wilson, 2011). DCs also release IL1β, IL6, IL23, and TGF-β to control Th17 cell differentiation (Terhune et al., 2013) and release IL27 to expand and activate CD8^+^ T cells, NKT cells, and Th1 cells, but suppress DCs and Th17 cells (Mascanfroni et al., 2013; Wei et al., 2013; Iwasaki et al., 2015; Huang et al., 2019; Figure 5B). DC expression of membrane-bound co-stimulatory molecule OX40 ligand (OX40L) provides a critical signal for Th2 survival, proliferation, activation, and cytokine expression (Jenkins et al., 2007; Okoye and Wilson, 2011). DCs also express co-stimulatory molecules Jagged and Delta-like ligands (DLL) that bind to the Notch receptors on T cells to control the differentiation of T-cell subtypes including Th1, Th2, Th9, Th17, and Treg cells (Tindemans et al., 2017; Sun et al., 2018; Figure 5C). Therefore, DC differentiation directly controls the differentiation of various T-cell subtypes as an indirect mechanism to contribute cardiac remodeling after pressure overload injury. For example, Th1 cells promote cardiac fibroblast fibrosis (Nevers et al., 2017) whereas Th2 cells regulate B cell-mediated humoral responses against extracellular pathogens in addition to secrete Th2 cytokines IL4, IL5, IL10, and IL13. We recently showed that IL4 protected mouse cardiomyocytes from H_2_O_2_-induced apoptosis (Liu et al., 2020). As discussed, induced DC depletion in CD11c^+^DOG mice decreased the expression of profibrotic molecules collagen and connective tissue growth factor in hypertrophic heart (Araos et al., 2019), supporting a role for DCs in cardiac fibrosis. Besides the role of DCs in promoting T-cell activation as a mechanism to activate fibroblasts (Nevers et al., 2017), DCs also promote myofibroblast proliferation, differentiation, and activation (Chia et al., 2012), providing additional mechanisms of DC activity in profibrotic protein expression.

As an essential growth factor that drives DC differentiation (Rossetti et al., 2010), G-CSF improves cardiac function in mice after pressure overload-induced cardiac hypertrophy. G-CSF-treated mice showed increased cell adhesion molecule VCAM-1 and stem cell factor, reduced cardiac fibrosis and cardiac cell apoptosis, reduced LV posterior wall thickness, and increased cardiac cell post-surgery survival (Huber et al., 2015). A similar study was carried out by giving G-CSF to TAC-treated mice at different times: first 5 days after TAC injury or days 15 to 19. Mice were characterized at 28 days after TAC. Either treatment increased LV mass-to-BW ratio and LV anterolateral wall thickness, but reduced cardiac fibrosis and repaired cardiac function and remodeling at the 28-day time point (Li et al., 2012). Yet, these studies may not test a specific role for DCs in hypertrophic heart because the pleiotropic growth factor G-CSF also acts on other hematopoietic cells (Bendall and Bradstock, 2014). Therefore, prior studies did not test whether G-CSF activity in DC differentiation played any role in mice or humans receiving G-CSF treatment (Li et al., 2012; Huber et al., 2015; Farhadfar et al., 2020).

## Eosinophils

Eosinophils are a subset of circulating innate immune cells that contribute to cardiac cell death and myocardial fibrosis through their abundant mediators, such as TGF-β, EOS cationic protein (ECP), EOS-derived neurotoxin (EDN), major basic protein (MBP), EOS peroxidase (EPO), lysosomal hydrolytic enzymes, EOS peroxidase, and type 2 cytokines (Jacobsen et al., 2012; Rosenberg et al., 2013; Liu et al., 2020; Toor et al., 2020). Blood EOS counts serve as a risk factor for human cardiac diseases such as MI (Kirkeby and Paudal, 1960; Hällgren et al., 1979). EOS were found in autopsy specimens of patients with cardiac rupture post-MI (Atkinson et al., 1985) and in atherectomy specimens from patients with in-stent stenosis (Rittersma et al., 2006). Interestingly, low blood EOS count independently predicts cardiovascular death and correlates negatively with death rates (Cikrikcioglu et al., 2012).

**FIGURE 6:**
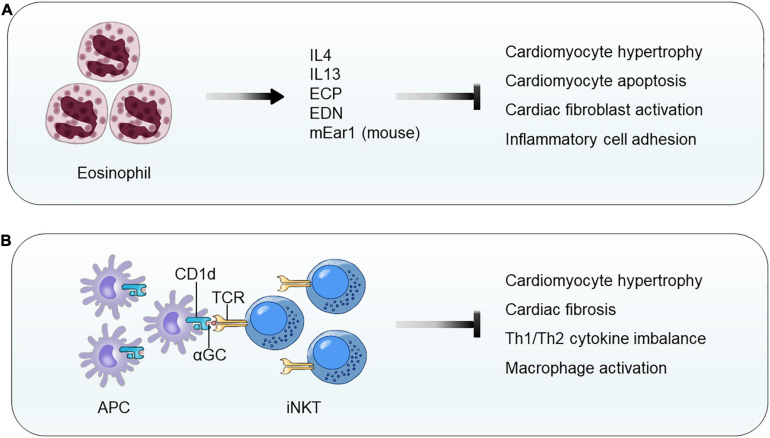
Eosinophil and invariant natural killer T-cell function in pressure overload-induced cardiac hypertrophy and fibrosis. **(A)** EOS release cytokines (IL4 and IL13) and cationic proteins (e.g., human ECP, human EDN, and mouse mEar1) to block cardiomyocyte hypertrophy and apoptosis, cardiac fibroblast activation, and inflammatory cell adhesion and migration. **(B)** CD1d-αGC-mediated activation of iNKT cells blocks cardiomyocyte hypertrophy and apoptosis, Th1/Th2 cytokine imbalance, and macrophage activation.

Although a direct evidence of EOS participation in cardiac hypertrophy is not publicly available, two independent studies demonstrated EOS functions in cardiac repair after MI injury. In patients with chest pain, blood EOS counts reduced within the first 12 h. In mice with experimental MI, blood and infarct region EOS counts increased over time. EOS genetic deficiency or antibody-mediated EOS depletion worsened the cardiac functions post-MI along with increased infarct size and myocardium fibrosis, reduced myocardium Th2 cytokines (IL4, IL10, and IL13), and increased myocardium chemokine C-X-C motif ligands (CXCL1 and CXCL2) and inflammatory cells (neutrophils and macrophages) at 4 days post-MI. Intraperitoneal administration of mouse recombinant IL4–anti-IL4 antibody complex did not improve heart function in WT mice, but significantly improved heart function in EOS-deficient *ΔdblGATA* mice (Toor et al., 2020). We reported similar but much more in-depth analyses of EOS function in cardiac remodeling post-MI. Mouse and human EOS protected H_2_O_2_-induced mouse cardiomyocyte death, blocked TGF-β-induced mouse cardiac fibroblast Smad2 and Smad3 activation, and reduced TNF-α-induced neutrophil adhesion on endothelial monolayer. Mechanistic studies showed that exacerbated cardiac dysfunction post-MI in EOS-deficient *ΔdblGATA* mice can be fully recovered by giving mice EOS cationic protein mEar1 (mouse EOS-associated-ribonuclease-1) or adoptive transfer of EOS from WT mice, but not those from IL4-deficient (*Il4^–/–^*) mice, suggesting a role for EOS-derived mEar1 and IL4 in repairing cardiac injury. In cultured mouse cardiomyocytes, *Il4^–/–^* EOS or EOS pretreated with mEar1 antibody failed to block H_2_O_2_-induced cell death. Although recombinant IL4 did not affect TGF-β-induced Smad2 and Smad3 signaling, EOS from WT, *Il10^–/–^*, and *Il13^–/–^* mice but not EOS from *Il4^–/–^* mice effectively blocked TGF-β-induced Smad2 and Smad3 activations in mouse cardiac fibroblasts. Human EOS acted, similarly. Human EOS isolated from human blood dose-dependently blocked hypoxia-induced apoptosis of primary cultured human cardiomyocytes and TGF-β-induced Smad2 and Smad3 signaling in primary human cardiac fibroblasts (Liu et al., 2020; Figure 6A). Although a direct test of EOS activity in pressure overload-induced cardiac hypertrophy remains unavailable, studies from mouse MI models (Liu et al., 2020; Toor et al., 2020) suggest that EOS also use IL4 and cationic proteins to block cardiomyocyte death and to control cardiac fibroblast activation.

## Invariant Natural Killer T Cells

iNKT cells, a subset of T lymphocytes, are an innate-like T lymphocyte population that recognizes the glycolipid ligands presented from antigen-presenting cells by CD1d, a non-polymorphic MHC-class I-like molecule (Crosby and Kronenberg, 2018). In TAC-induced mouse cardiac hypertrophy, myocardium iNKT cells increased by three- to fourfold. Deficiency of iNKT cells in *Jα18^–/–^* mice increased LV end systolic and diastolic diameters, reduced LV FS, and increased LV mass. Histological and immunoblot analyses demonstrated that iNKT cell deficiency increased myocardium cardiomyocyte hypertrophy, cardiac fibrosis, and myocardium ERK signaling. iNKT cell deficiency also led to an imbalance of myocardium Th1 and Th2 cytokines with significantly reduced IL10, IFN-γ, and TNF-α (Takahashi et al., 2020; Figure 6B).

Use of CD1d-deficient mice indirectly proved a role for iNKT cell in cardiac hypertrophy (Wang et al., 2019a). Mice receiving Ang-II infusion showed cardiac dysfunction (significantly increased EF and FS), increased HW and cardiomyocyte size and expression of hypertrophic molecules ANP and BNP, increased cardiac fibrosis and collagen I and III expression, elevated TGF-β expression and Smad2 and Smad3 activation, and enhanced myocardium macrophage accumulation, NF-κB activation, and inflammatory cytokine expression (IL1β and TNF-α). All these pathological changes became worse in CD1d-deficient mice, but significantly improved when iNKT cells in WT mice were activated by treating mice with α-galactosylceramide (αGC) (Figure 6B). WT mice receiving bone marrow transplant (BMT) from CD1d-deficient mice displayed the same phenotypes as the CD1d-deficient mice in Ang-II infusion-induced cardiac dysfunction, hypertrophy, and remodeling. In Ang-II infusion-induced cardiac hypertrophy, administration of recombinant IL10 to CD1d-deficient mice fully reversed the adverse phenotypes of these recipient mice (Wang et al., 2019a). Therefore, like EOS, iNKT cells are another cardioprotective cell type that prevents or mitigates cardiac hypertrophy, cardiac remodeling, and HF.

## Conclusion and Future Prospects

In conclusion, innate immune cells play essential roles in the initiation and progression of cardiac hypertrophy and fibrosis. A great number of studies have focused on various innate immune cell types and their molecules and regulators that are associated with cardiomyocyte hypertrophy and cardiac fibrotic remodeling after the onset of pressure overload. The accumulation of these cells in the atrium or ventricles occurs immediately after the initiation of injury until late phases. Yet, their cardioprotective or cardiodestructive activities can differ depending on the cell types, subtypes, secretory molecules, and time courses of myocardium infiltration. In general, EOS and iNKT cells display cardioprotective activities, but MCs, neutrophils, and DCs exert detrimental functions on cardiomyocytes and cardiac fibroblasts. Macrophages and monocytes possess two sides of influences, promoting the pathological development or negatively regulating cardiac hypertrophy and remodeling, depending on their expression of Ly6C and CD206 in addition to their different surface chemokine receptors. Pressure overload stimulates the secretion of cytokines, metabolites, or growth factors from innate immune cells and resident cardiomyocytes, which together mediate innate immune cell infiltration into the heart. In turn, these cells affect cardiomyocyte pro-hypertrophic pathways and cardiac fibroblast activation.

At the cellular levels, we can use different approaches to add, remove, or regulate the population or activities of each discussed cell type. Yet, the mechanisms by which each innate immune cell participates in pressure overload-induced cardiac hypertrophy and fibrosis have not been fully understood. The hypertrophic or pro-fibrogenic signaling modulated by innate immune cells or their granules and secretories remains unknown. The heterogeneity of different innate immune cell types and subsets provides an explanation for the functional complexities in the process of this cardiac disease. For instance, macrophage and monocyte heterogeneity alone exerts opposite functions depending on the cellular subtypes (Figure 3). Neutrophil expression of MMP and S100a8/a9 may reduce ECM accumulation and block cardiomyocyte hypertrophy and cardiac fibroblast pro-collagen expression, although these cells were proven cardiodestructive (Figure 4). Similarly, cDCs and pDCs may play opposite roles in cardiac fibrosis and cardiac cell death, although there is currently no study to test their functional differences (Figure 5). To date, EOS and iNKT behaved differently from MC, neutrophils, and DCs in hypertrophic heart or other relevant cardiac diseases such as MI. Indirect evidence supports a cardioprotective role for EOS and iNKT in cardiac hypertrophy and fibrosis (Figures 6A,B). Yet, it is always possible that future studies may find that some molecules from these cells might exert a cardiodestructive activity. Therefore, new techniques such as lineage-specific genetic tools might be helpful to analyze their specific clusters and precise roles. Furthermore, single-cell sequencing has emerged as a rapidly developing frontier technology for cellular research. This technology might make it possible to identify innate immune cell heterogeneities and reveal their different cellular kinetics at the single-cell level.

Studies of pressure overload-induced hypertrophy and fibrosis were mostly performed in experimental animals with single genetic background or sex. The genetic background complexities of humans are much more sophisticated than experimental animals. Recent clinical studies support an essential role of inflammation in human cardiovascular diseases. Antibodies against IL1β and IL6 successfully reduced the systemic inflammation and cardiovascular events (Ridker et al., 2017; Ridker et al., 2021). Studies of innate immune cells in experimental models of pressure overload may benefit patient experiencing chronic pressure overload, such as those with HFpEF or hypertension (Mishra and Kass, 2021). Because of the complexities of macrophages, monocytes, and DC subsets, and functional differences of neutrophils at different stages after pressure overload, direct and nonselective targeting of these cells may not benefit patients. Yet, indirect evidence or preliminary studies of MCs, EOS, and iNKT cells in experimental models of pressure overload, HF, or heart infarction suggest the possibility to benefit patients by blocking MC degranulation, targeting MC proteases, using EOS-specific mediators such as EOS cationic protein, and activating iNKT cells with αGC. Therefore, with a better understanding of innate immune cells and their mechanisms regarding pressure overload-induced cardiac remodeling, development of therapeutic strategies to target innate immune cells as novel approaches to attenuate hypertensive cardiac hypertrophy and dysfunction in patients under chronic pressure overload may become possible.

## Author Contributions

XL wrote the draft and made the figures. G-PS edited the manuscript and designed the figures. JG helped edit the manuscript and the figures. All authors contributed to the article and approved the submitted version.

## Conflict of Interest

The authors declare that the research was conducted in the absence of any commercial or financial relationships that could be construed as a potential conflict of interest.

## Publisher’s Note

All claims expressed in this article are solely those of the authors and do not necessarily represent those of their affiliated organizations, or those of the publisher, the editors and the reviewers. Any product that may be evaluated in this article, or claim that may be made by its manufacturer, is not guaranteed or endorsed by the publisher.
